# Areal parameter estimates from multiple datasets

**DOI:** 10.1098/rspa.2019.0352

**Published:** 2019-11-06

**Authors:** B. L. N. Kennett

**Affiliations:** Research School of Earth Sciences, The Australian National University, Canberra ACT 2601, Australia

**Keywords:** spatial interpolation, multiple datasets, data fusion

## Abstract

A wide range of methods exist for interpolation between spatially distributed points drawn from a single population. Yet often multiple datasets are available with differing distribution, character and reliability. A simple scheme is introduced to allow the fusion of multiple datasets. Each dataset is assigned an *a priori* spatial influence zone around each point and a relative weight based on its physical character. The composite result at a specific location is a weighted combination of the spatial terms for all the available data points that make a significant contribution. The combination of multiple datasets is illustrated with the construction of a unified Moho surface in part of southern Australia from results exploiting a variety of different styles of analysis.

## 1. Introduction

Many procedures for constructing surface representations of geophysical information are based on the assumption that the available samples are drawn from a single stochastic population. Such concepts underlie kriging methods (e.g. [1]) and their extension to Gaussian processes (e.g. [2]). Other spatial interpolation schemes are designed to generate a surface that passes directly through a sparse set of sample points, with assumptions about the fitting surface such as minimum curvature [3]. A useful summary of many different methods of spatial interpolation and their limitations is provided in [4].

However, in some circumstances, there can be multiple spatial estimates for a parameter with different distributions and reliabilities, e.g. from surveys carried out at various times with different equipment. It is possible to compensate to some extent by introducing relative weighting between data points in a single mode of interpolation.

Although registered at a point, most geophysical datasets sample a zone around the observation point whose size will vary with the technique employed. The situation becomes even more complicated when data estimates are based on different physical approaches, as occurs, e.g. in the representation of the depth to Moho. A number of methods provide information on the crust-mantle boundary such as the interpretation of refraction and reflection surveys, receiver functions and autocorrelation techniques. Multiple methods may also be used at the same seismic stations.

The approach used in [5] to construct a Moho surface across the Australian continent was to assign specific weights to each observation, and combine all results in a 0.5° × 0.5° pixel with the weighted average assigned to centre of each pixel. These new values were then used as the basis for interpolation using an adjustable tension continuous curvature gridding algorithm [3]. These data averaging suppresses the local information present in, e.g. full-crustal reflection profiling where independent estimates of the depth to the base of the crust can be made every few kilometres. Thus, significant detail is suppressed to achieve a smooth fitting surface. Furthermore, when the only result in a pixel is assigned low weight, this value will be transmitted directly into the continent-wide surface.

Since 2011 the number of Moho estimates in Australia has grown significantly, with a strong continuing programme of full-crustal reflection profiling and the advent of new methods such as the exploitation of stacked station auto-correlograms [6,7]. In many places this means that there are multiple values for the depth to Moho based on different physical assumptions.

Here, we propose a new scheme in which we take account of the nature of different classes of observation, not only with data weights but with varying spatial influence zones. The relative weighting of different styles of datasets can also be adjusted. The resulting expression for the data estimate at any spatial point has a formal similarity to kriging and other single dataset interpolators, but combines results from multiple datasets.

## 2. Exploiting multiple datasets

With a suite of datasets {*d*}, we assign internal weights *w*_*kd*_ to each data point *m*_*kd*_ in dataset *d*. All points in the same dataset are then assigned a spatial influence depending on the nature and reliability of the data. We use radial Gaussian spread functions with a scale length *σ*_*d*_ to describe the spatial patterns, building on the work of [6] who used such weighting for spatial stacking of data from many seismic stations in southeastern Australia.

We combine the information from different datasets allowing for relative weighting between data sets specified by a weight *w*_*d*_ that is dataset specific. We then build the estimate of the local value of the parameter at the *i*th spatial point *M*_*i*_ from a sum of all contributions with allowance for weighting and the distance of the data points from the sample point:

2.1 $$M_{i}=\frac{M_{i}^{w}}{W_{i}^{w}}=\frac{\sum_{d}w_{d}\sum_{k}w_{kd}m_{kd}e^{-{\left(\Delta_{ik}/\sigma_{d}\right)}^{2}}}{\sum_{d}w_{d}\sum_{k}w_{kd}e^{-{\left(\Delta_{ik}/\sigma_{d}\right)}^{2}}},$$

where Δ_*ik*_ is the distance from the *i*th sample point to the observation. In principle, all data points contribute to an estimated parameter value, but it is expedient to truncate each spatial spread at a spatial separation of 3.5*σ*_*d*_ to avoid cumulation of numerical truncation error from exceedingly small weights. With this truncation level the smallest areal weight will be 5 × 10^−6^, so that calculations can be carried out in single precision arithmetic.

The resulting formula for the spatial estimate has comparable structure to interpolation with inverse distance weighting [8] or kriging [1] but the weights are determined *a priori* based on the nature of each measurement rather than being determined by the data point distribution. The representation (2.1) also allows for multiple data classes with their own character, rather than assuming all points are drawn from a single random variable distribution as in kriging. For example, data values from diverse distributions may be the result of very different spatial sampling and hence have no intrinsic relation to each other.

**Figure 1.**
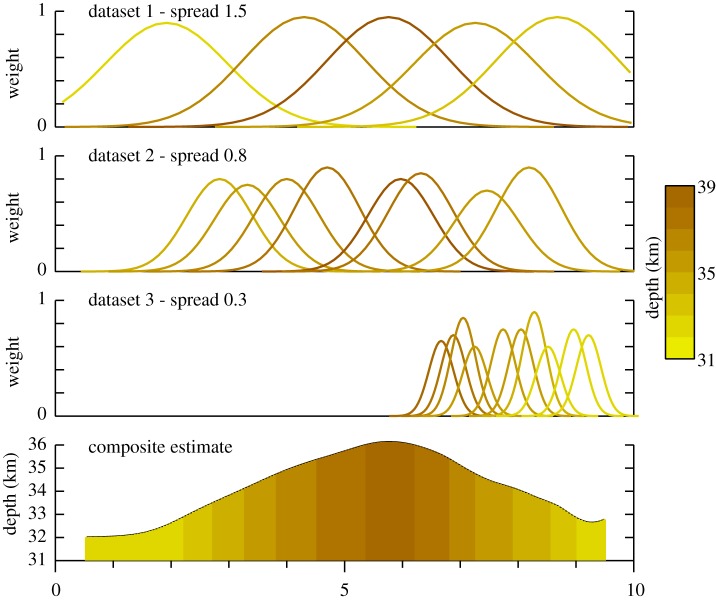
Application of weighted spatial-spread method to three distinct datasets with variable spatial influence and reliability, as indicated on the top three rows, to produce a single spatial surface shown on the bottom line. The data points are colour coded by their value. The spatial extent and weight for each is drawn directly. (Online version in colour.)

The concept of the weighted spatial-spread procedure is illustrated in figure 1, where we show the construction of a single surface from a combination of three datasets. The first dataset comprises a limited number of data points with high reliability that have broad spatial sampling; the second dataset has medium quality and spread with greater variation in reliability and the third rather dense data of variable quality and narrow spatial influence. In the upper three rows of figure 1, we plot the spatial-spread assigned to each data point colour coded by the actual data value. The bottom row shows the resulting distribution when all the data points are combined using the weighted spatial-spread formula (2.1) at a dense line of sample points. In this simple example, each distinct dataset is assigned equal weight, and only the internal weights are taken into consideration.

The weighted spatial-spread result (2.1) is simple to implement and is very versatile. The estimator may be made with absolute data values or in relative mode where one examines deviations from a reference value or a predefined reference surface. When used with just a single dataset it can provide a smoothing interpolator with the degree of smoothness controlled by the assigned spatial spread. Alternatively, one can represent spatially distinct points with combination of results only where the influence zones overlap.

We can examine the consistency of the various datasets by constructing a variance estimate at each sample location, using the weighted value for the square of the samples, and the square of the sample estimate *M*_*i*_:

2.2 $$S_{i}=\frac{\sum_{d}w_{d}\sum_{k}w_{kd}m_{kd}^{2}e^{-{\left(\Delta_{ik}/\sigma_{d}\right)}^{2}}}{\sum_{d}w_{d}\sum_{k}w_{kd}e^{-{\left(\Delta_{ik}/\sigma_{d}\right)}^{2}}}-M_{i}^{2}.$$

The measure $\sqrt{S_{i}}$ provides a convenient summary of the deviation from the sample estimate *M*_*i*_. A further useful quantity is a weighted combination of the uncertainty estimates for the individual data points, constructed in the same way as in (2.1).

The nature of the expression (2.1) is such that it is possible to add in the effect of a new dataset, without recomputing from scratch, provided that the quantities *M*^*w*^_*i*_, *W*^*w*^_*i*_ have been stored. With the additional dataset, the revised value of *M*_*i*_ is

2.3 $$M_{i}=\frac{M_{i}^{w}+w_{d}\sum_{k}w_{kd}m_{kd}e^{-{\left(\Delta_{ik}/\sigma_{d}\right)}^{2}}}{W_{i}^{w}+w_{d}\sum_{k}w_{kd}e^{-{\left(\Delta_{ik}/\sigma_{d}\right)}^{2}}},$$

exploiting the values *m*_*kd*_ and weights *w*_*kd*_ for the new data. A similar approach can be used for the variance estimate.

## 3. Application: multiple Moho datasets

**Figure 2.**
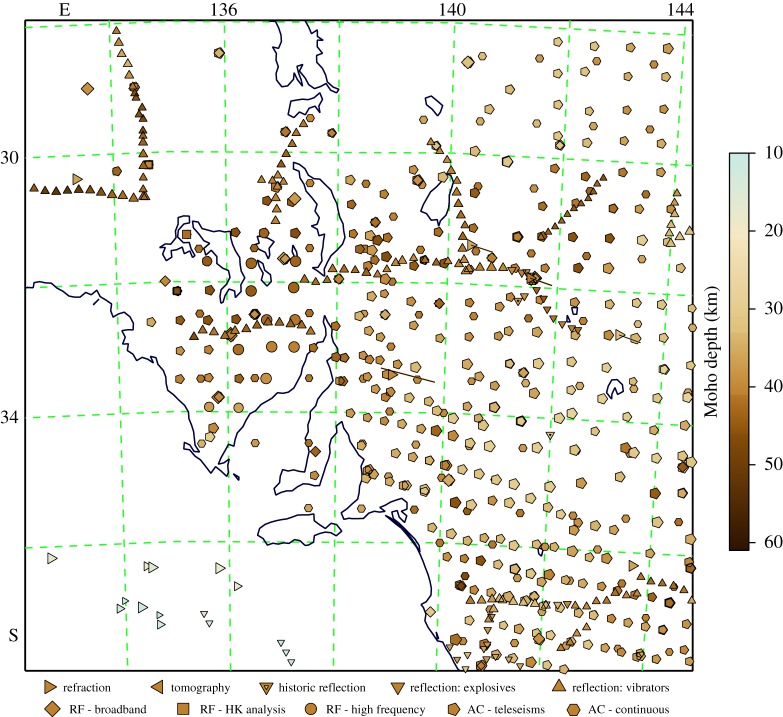
Distribution of Moho estimates across part of southern Australia. The points are colour-coded by depth with symbol shape determined by measurement type, scaled by reliability. (Online version in colour.)

As an example of the application of the weighted spatial-spread approach we present results for estimates of the depth to Moho for an area of southern Australia. This region has a broad range of different data samples with an uneven data distribution. In figure 2, we show the full range of data points. The points are colour coded by Moho depth with symbols that indicate the nature of the measurement, and scaled to indicate the reliability of the results and thus the weights applied.

**Figure 3.**
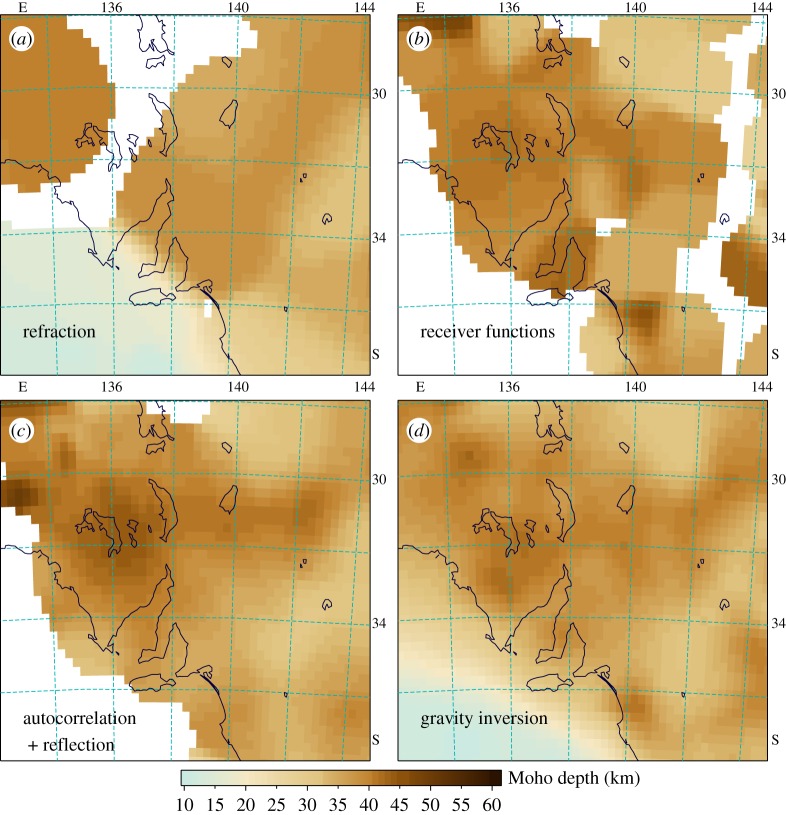
Estimates of Moho depth using the weighted spatial spread approach for restricted data sets: (*a*) refraction and marine wide-angle information; (*b*) receiver functions; (*c*) autocorrelation and reflection profiling and (*d*) gravity inversion. (Online version in colour.)

**Figure 4.**
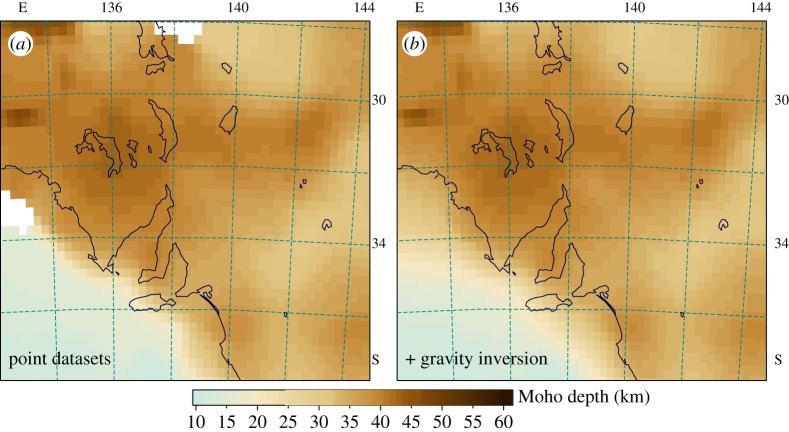
Estimates of Moho depth using the weighted spatial spread approach: (*a*) all nine sets of point data and (*b*) with the inclusion of a low weight applied to gravity inversion.

In table 1, we show the relative weight assigned to the datasets and the spatial spreads used in creating figures 3 and 4. The spread estimates are based on the spatial sampling associated with each data class. Thus refraction experiments average over more than 100 km, whereas receiver-based techniques are more localized. Results from full-crustal reflection surveys are available at much closer spacing, but show more variation in data quality and involve uncertainty in conversion between reflection time and depth. The spatial spread assigned to reflection data is designed to preserve such features as abrupt changes in crustal thickness (cf. [9]).

**Table 1.** Weighting used for multiple datasets.

data type	weight	spatial spread [°]
refraction	1.0	1.2
marine wide-angle	1.0	0.4
receiver functions	1.0	0.6
tomography	1.0	0.6
autocorrelation	0.9	0.5
reflection	0.9	0.2
gravity inversion	0.5	0.4

In addition to these point estimates of crustal thickness, we have made use of the gravity inversion of [10] using samples on a 0.5° × 0.5° grid. These inversion results are assigned a uniformly low weight (0.4) within the dataset. Figure 3 shows the results for each of the major classes of data with truncation of the estimates at the same weighting threshold (0.02). None of the point data achieves full coverage of the region. Even when all this data is combined there are some patches where no direct estimation can be made, and considerable areas where low weight results have to be employed (figure 4*a*). Although the receiver function results are given high weight, the broadband stations employed are well dispersed and their limited number does not have as strong an influence on the final result as the autocorrelation results from a much larger number of short-period stations and the same broadband stations.

When we add in the results from the gravity inversion we achieve a better rendering for the whole domain (figure 4*b*). We have used a weighting of 0.5 for the entire dataset, which coupled to the weighting of 0.4 for each data point, gives an effective weight for the contribution to the sample estimate of 0.2. Even with such a low weighting, the presence of the results from the gravity inversion has a large impact on the resolution of the continent-ocean transition, but also gives an improved result in areas with limited data coverage since it fills in the holes.

**Figure 5.**
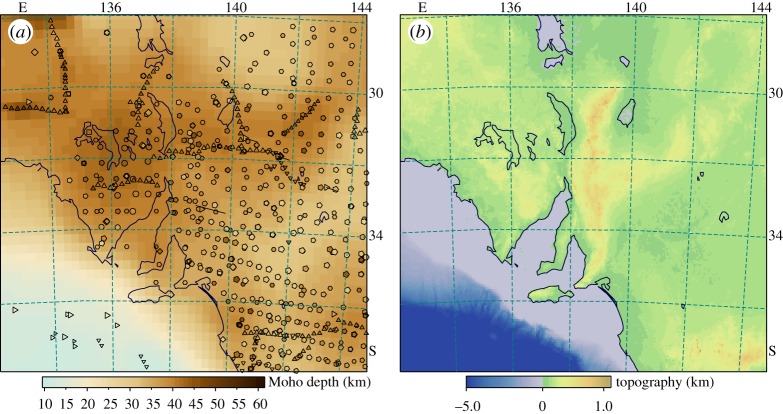
(*a*) Comparison of the Moho surface derived by combining all the various classes of information using the weighted spatial spread approach with the original data values. The symbols are marked as in figure 2, to distinguish the various classes of information; (*b*) the topography across the region. (Online version in colour.)

**Figure 6.**
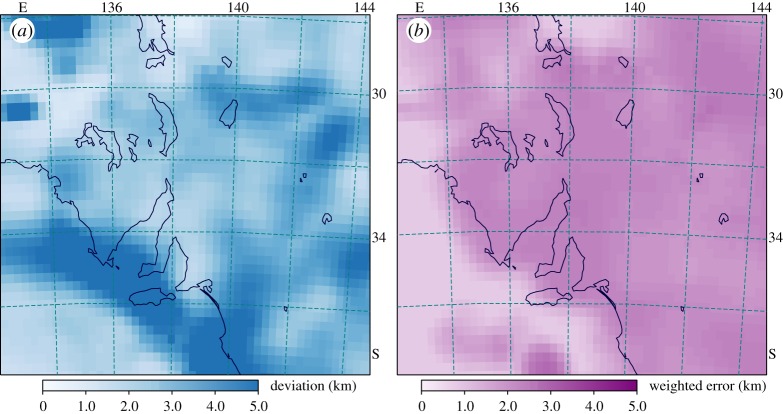
(*a*) Measure of consistency of Moho values using the $\sqrt{S_{i}}$ distribution. (*b*) Weighted uncertainty distribution. (Online version in colour.)

In figure 5*a*, we display the combination of all the different datasets and superimpose the original data values from figure 2. We note that a number of the autocorrelation estimates using earthquake signals remain discordant, even though they have influenced the construction of the Moho surface. The physical model used to extract these results enforces a sharp discontinuity [7], and the differences from the interpreted surface, where present, provide an indication of the thickness of the crust-mantle transition. We also display in figure 5*b* the topography across the region, which is relatively subdued. The patterns of Moho variation do not show a direct correlation with the topography. The lack of isostatic equilibrium is reflected in neotectonic activity in the Flinders Ranges (the elevation along 138° E) that have no deep-seated root. Whereas, the thicker crust is found in the cratonic zones that show little surface topography, In figure 6, we display the spatial distribution of the measure of data consistency $\sqrt{S_{i}}$ from (2.3) in (a), and an estimate of the geographical distribution of weighted uncertainty in data values in (b). For the Moho datasets, we do not have uncertainty measure for all points, but in every case have a quality proxy. We have used an estimate of uncertainty, in kilometres, using the empirical relation

3.1 $$e_{kd}=0.8+6.0*(1.0-w_{kd}),$$

calibrated against receiver function results. We construct a weighted estimate, as in (2.1). at each sample point *i* using *e*^2^_*kd*_ and take the square root for display in figure 6*b*.

The main zone of inconsistency in Moho estimates (figure 5*a*) comes at the continental margin where both continental and oceanic point values influence the combination with low weights. The inclusion of the gravity inversion results, resolves the ocean-continent transition well (figure 4*b*), but the discordant influences remain. Other patches where consistency issues arise occur where dense seismic reflection results are introduced into a region where other information is limited, when the ‘deviation’ length approaches 10% of the Moho estimate. Elsewhere the consistency is generally better than 5% of the Moho value. The consistency patterns do not show any strong correlation with the weighted measure of measurement uncertainties (figure 6*b*). The discrepancies arise between different physical techniques for extracting Moho values, rather than the quality of individual measurements.

## 4. Discussion and conclusion

We have established a procedure that enables multiple datasets to be combined to produce a single spatial surface, together with a measure of data consistency. The approach is simple to implement. A very simple loop structure is needed to produce the sample estimate, once the spherical distance from the sample value to the data points has been calculated. The weighted spatial-spread procedure (2.1) produces a very flexible scheme for estimating a parameter surface, since the character of the combination is under full user control through *a priori* choices for the weighting of data points and the spatial spread around every point assigned to each dataset. This means that different choices for the relative weighting of different datasets can readily be explored. Indeed it is possible to look at the uncertainties associated with the inclusion of particular datasets by setting up an ensemble of surface constructions.

It is desirable that the spatial influence zone assigned to each class of observation conforms to the physical nature of the actual measurement, rather than be arbitrarily extended. The use of such confined influence zones may mean that parameter estimates are more spatially restricted than in a standard broad-scale interpolation, but they will represent the direct impacts of the measurements. In this context, it can be useful to employ a background field that can be assigned low weight to compensate for data holes, as in the use of the gravity inversion results in the Moho results in the example.

In the treatment above, we have used radial Gaussian spread functions around each data point, but these functions could be replaced by other spatial samplers if they are more appropriate to different classes of information. For data that has a strongly directional character it may well be worthwhile to use a spatial influence zone with an elliptical footprint defined by two scale lengths and a preferred axis of orientation. If slower spatial decay is desired then a simple exponential of spatial separation can replace the quadratic form.

The weighted spatial-spread approach also works well for sparse observations, such as heat flow, that characterize a limited spatial zone. As with the restricted datasets in figure 3, the results will have blank zones, but this is preferable to long-distance extrapolation of data that reflect local conditions.

## Supplementary Material

Source code

## Data accessibility

The Moho data values are lodged at the Australian seismological data repository (auspass.edu.au/research/AusMoho.html) as a set of ascii files. A Fortran code implementing this approach is available in the electronic supplementary material accompanying this article.
