# Supplementary material for: Areal parameter estimates from multiple datasets
Source: Proc Math Phys Eng Sci. 2019 Nov 6;475(2231):20190352. doi: 10.1098/rspa.2019.0352 (PMC6894539; doi:10.1098/rspa.2019.0352)
Supplement: Source code [file rspa20190352supp1.pdf]

Supplement to:

Areal parameter estimates from multiple datasets

B.L.N Kennett

Research School of Earth Sciences, The Australian National University, Canberra ACT 2601, Australia

Implementation of weighted spatal-spread procedure

The extration of a single parameter surface from a suite of data sets corresponding to different measurement techniques with varying spatial spreads and individual data weights has been implemented in a simple Fortran-77 code.

The Moho data used in the paper can be downloaded from
http://auspass.edu.au/research/AusMoho.html
Doi: 10.25911/5cf751c17b3d4

The source code on the following pages has been compiled with gfortran under MacOSX

```
gfortran -o bin/GHMoho3.x  GHmoho3.f
```

and should be run as, e.g.,

```
bin-GHMoho3.x < GH3.in
```

A sample input file is provided, which sepcifies the data sets to be used and the doamin in which the model estimates are produced.
The output is a set of three grid files, e.g.,

Model parameter grid:	`GHM3test.xyzv`
Consistency grid:	`GHM3test.xyzs`
Weighted error grid:	`GHM3test.xyze`

The format of these files is plain text, with values registered to latitude and longitude

Latitude	Longitude	value	Mw	Ww
-39.0000	131.0000	11.53	6.49	0.56
-39.0000	131.2500	11.49	6.42	0.56
-39.0000	131.5000	11.49	4.10	0.36
-39.0000	131.7500	11.43	6.35	0.56
-39.0000	132.0000	11.40	6.33	0.56
-39.0000	132.2500	11.38	6.32	0.56
-39.0000	132.5000	11.37	6.34	0.56
-39.0000	132.7500	11.39	6.39	0.56

Mw is the sum of the weighted contributions, Ww the sum of the weights
The model value = Mw/Ww.

The grid files should be suitablefor use with many different mapping environments.

Source code:

```
C-----------------------------------------------------------------------
C       GHmoho3:    composition of multiple Moho estimators
C
C       Output to .xyz files
C       specified grid interval
C
c       gfortran -o GHMoho3.x  GHmoho3.f
C
C       B.L.N. Kennett RSES,ANU February/September 2019
C-----------------------------------------------------------------------
c
      character*40 cfile,cinput
      character*8 qfile
      character*4 csta
      real glat(200),glon(300)
      real xmoh(200,300),vmoh(200,300),emoh(200,300)
      real tmo(200,300),wmo(200,300),tm2(200,300),em2(200,300)
      real slat(20000),slon(20000),smoh(20000),sw(20000)
      real snlat,sslat,swlon,selon
      real xlat,xlon,dgrid,angsl,rwey
      integer nglon,nglat,kq
c
      write(6,*) "file containing list (12 chars)"
      read(5,*) cfile
      write(6,*) cfile
      write(6,*) "name of output - 8 chars"
      read(5,*) qfile
c set up grid
      write(6,*) "south latitude, north latitude"
      read(5,*) sslat, snlat
      write(6,*) "west longitude, east longitude"
      read(5,*) swlon, selon
      write(6,*) "grid interval"
      read(5,*) dgrid
c
      nglat = int((snlat-sslat)/dgrid)+1
      nglon = int((selon-swlon)/dgrid)+1
      write(6,*)  nglat,nglon
      xlat = sslat
      xlon = swlon
      do i=1,nglat
        glat(i) = xlat
        xlat = xlat+dgrid
      enddo
      do j=1,nglon
        glon(j) = xlon
        xlon = xlon+dgrid
      enddo
c
      do i=1,nglat
        do j=1,nglon
          tmo(i,j) = 0.0
          wmo(i,j) = 0.0
          xmoh(i,j) = 0.0
        enddo
      enddo
c
      open(10,file=cfile,status='old')
      read(10,*) nfile
      DO N = 1,nfile
        read(10,fmt='(a)') cinput
```

```
          read(10,*)  rwey, angsl
          write(6,*)  rwey, angsl, cinput
          open(11,file=cinput,status = 'old')
          read(11,*)
          kq=1
          do k=1,16000
            read(11,end=19,fmt="(8x,2f12.4,2x,2f10.2)")
     ^          slat(k),slon(k),smoh(k),sw(k)
            kq = kq+1
          enddo
19        write(6,*) kq
          close(11)
c                      scan across area
          do i = 1,nglat
            do j = 1,nglon
              do k=1,kq
                call ydiz(glat(i),glon(j),slat(k),slon(k),
     ^                  delta,deltak)
                qs = delta
                if(qs.lt.3.6*angsl) then
                  aqs = (qs*qs)/(angsl*angsl)
                  qw = exp(-aqs)
                  wmm = sw(k)*qw*rwey
                  if(sw(k).gt.0.45) then
                     err = 0.8+6.0*(1.0-sw(k))
                  else
                     err = 0.8
                  endif
                  tmo(i,j) = tmo(i,j) + smoh(k)*wmm
                  tm2(i,j) = tm2(i,j) + smoh(k)*smoh(k)*wmm
                  em2(i,j) = em2(i,j) + err*err*wmm
                  wmo(i,j) = wmo(i,j) + wmm
                endif
              enddo
            enddo
          enddo
        ENDDO
c
        open(15,file=qfile//".xyzv")
        open(16,file=qfile//".xyze")
        open(17,file=qfile//".xyzs")
        do i = 1,nglat
          do j = 1,nglon
            if(wmo(i,j).ge.0.02) then
              xmoh(i,j) = tmo(i,j)/wmo(i,j)
              vv = tm2(i,j)/wmo(i,j)
              vmoh(i,j) = sqrt(vv-xmoh(i,j)*xmoh(i,j))
              emoh(i,j) = sqrt(em2(i,j)/wmo(i,j))
            else
              xmoh(i,j) = 0.0
              vmoh(i,j) = 0.0
              emoh(i,j) = 0.0
            endif
            write(15,fmt='(4x,2f10.4,2x,3f10.2)')
     ^            glat(i),glon(j),xmoh(i,j),tmo(i,j),wmo(i,j)
            write(16,fmt='(4x,2f10.4,2x,3f10.2)')
     ^            glat(i),glon(j),vmoh(i,j),tm2(i,j),wmo(i,j)
            write(17,fmt='(4x,2f10.4,2x,3f10.2)')
     ^            glat(i),glon(j),emoh(i,j),em2(i,j),wmo(i,j)
          enddo
        enddo

c
        stop
```

```
      end
      subroutine ydiz(clats,clons,clatr,clonr,
     &                delta,deltak)
cc----------------------------------------------------------------------
c                          Calculates distance
c                          for spheroidal earth between
c                          specified geographic source and
c                          receiver station coordinates
c YDAZ modified 2015
cc----------------------------------------------------------------------
c+                                     copyright B.L.N. KENNETT
c+                                     R.S.E.S. A.N.U. January 1978
cc---------------------------------------------------------------------
      implicit double precision (a-h,o-z)
      real clats,clons,clatr,clonr,delta,deltak
c                          radius on spheroid
      gra(x,y,e) = dsqrt( (1.0d0-e)**2 /
     &                 ((1.0d0-e*y)**2 + e*e*x*y ) )
      ecc = 0.003367
      re = 6378.388
      ec1 = (1.0d0-ecc)**2
      pi = 3.141592653589793
      pib2 = pi/2.0
      degr = pi/180.0
      dlats = clats*degr
      dlons = clons*degr
      dlatr = clatr*degr
      dlonr = clonr*degr
c                          geocentric coordinates
      aa=ec1*sin(dlats)
      bb=cos(dlats)
      glats = datan2 (aa,bb)
      glatr = datan2 ( ec1*sin(dlatr) ,1.0d0*cos(dlatr) )
      sps = sin(glats)**2
      cps = cos(glats)**2
      spr = sin(glatr)**2
      cpr = cos(glatr)**2
c                          radii at source,receiver
      rs = re*gra(sps,cps,ecc)
      rr = re*gra(spr,cpr,ecc)
c
      trs = pib2 - glats
      prs = dlons
      trr = pib2 - glatr
      prr = dlonr
c                          direction cosines for source
      AS = dsin(trs)*dcos(prs)
      BS = dsin(trs)*dsin(prs)
      CS = dcos(trs)
c                          direction cosines for receiver
      AR = dsin(trr)*dcos(prr)
      BR = dsin(trr)*dsin(prr)
      CR = dcos(trr)
c                          djstance
      cosdr = AS*AR + BS*BR + CS*CR
      deltar = dacos(cosdr)
c
      deltak = deltar*0.5d0*(rr+rs)
      delta = deltar/degr
c
      return
      end
```

Sample input file:

```
GHfiles3.lis                                       ! file list
GHM3test                                           ! name of output
-39.0   -27.0                                      ! south, north latitude
131.0   146.0                                      ! west, east latitude
0.25                                               ! grid interval
```

Sample file list:

```
10                                                 ! number of datasets
H-aitken.xyz                                       ! filename
0.5   0.4                                          ! spatial spread, weighting
H-refract.xyz
1.0   1.2
H-marine.xyz
1.0   0.4
H-RecfA.xyz
1.0   0.6
H-RecfB.xyz
1.0   0.6
H-RecfC.xyz
1.0   0.6
H-ACcont.xyz
0.9   0.5
H-ACteq.xyz
0.9   0.6
H-reflect.xyz
0.9   0.2
H-rtomo.xyz
1.0   0.6
```

Illustration of data file - first few lines of H-RecfA.xyz

Note presence of repeatedvalues (based on multiple separate estimates)

Format:

Name	latitude	longitude	value	weight

```
Receiver Functions A - Broad band waveform inversion
ARMA        -30.4198    151.6280        38.00       1.00
WOOL        -31.0730    121.6780        40.00       0.90
CAN         -35.3208    148.9986        49.00       0.80
CTAO        -20.0883    146.2544        40.00       1.00
WRAB        -19.9443    134.3409        50.00       0.90
TAU         -42.9099    147.3204        32.00       1.00
NWAO        -32.9270    117.2330        42.00       0.90
MBWA        -21.1600    119.8330        29.00       1.00
ARMA        -30.4200    151.6300        36.00       0.95
BBOO        -32.8100    136.0600        42.00       0.95
BLDU        -30.6100    116.7100        36.00       0.95
COEN        -13.9600    143.1700        38.00       0.95
CTAO        -20.0900    146.2500        40.00       0.95
```
